# ﻿A new species of *Leptobrachella* Smith, 1925 (Anura, Megophryidae) from the coastal forest of Dak Lak Province, Vietnam

**DOI:** 10.3897/zookeys.1267.177118

**Published:** 2026-01-20

**Authors:** Dang Trong Do, Truong Quang Nguyen, Chung Van Hoang, Thomas Ziegler, Cuong The Pham

**Affiliations:** 1 Faculty of Natural Sciences, Phu Yen University, Dak Lak Province, Tuy Hoa Ward, Vietnam University of Cologne Cologne Germany; 2 Institute of Biology, Vietnam Academy of Science and Technology, 18 Hoang Quoc Viet Road, Hanoi 10072, Vietnam Institute of Biology, Vietnam Academy of Science and Technology Hanoi Vietnam; 3 Graduate University of Science and Technology, Vietnam Academy of Science and Technology, 18 Hoang Quoc Viet Road, Hanoi 10072, Vietnam Phu Yen University Tuy Hoa Ward Vietnam; 4 Cologne Zoo, Riehler Straße 173, 50735, Cologne, Germany Graduate University of Science and Technology Hanoi Vietnam; 5 Institute of Zoology, University of Cologne, Zülpicher Straße 47b, 50674, Cologne, Germany Cologne Zoo Cologne Germany

**Keywords:** Asian Leaf-litter toads, Deo Ca Mountain, genetic divergence, *Leptobrachella deocaensis* sp. nov., morphology, taxonomy

## Abstract

A new species of *Leptobrachella* is described from the coastal forest of Deo Ca Mountain in Dak Lak Province, Vietnam, based on morphological differences and genetic divergences in 16S rRNA mitochondrial gene sequences. The new species is distinguished from other species of the genus *Leptobrachella* by body size, dorsal skin texture, absence of ventrolateral and femoral glands, absence of lateral fringes on fingers and toes, color pattern of head and body, and iris color. The new species is divergent from other congeners by at least 6.34% uncorrected genetic distance (16S rRNA gene). *Leptobrachella deocaensis***sp. nov.** is genetically closest to *L. macrops* from Vietnam, with strong nodal support from both BI and ML analyses (1.00/98).

## ﻿Introduction

Asian Leaf-litter toads were historically assigned to several genera, including *Nesobia* Van Kampen; *Leptobrachella* Smith; *Paramegophrys* Liu; *Leptolalax* Dubois; *Lalax* Delorme, Dubois, Grosjean & Ohler; and *Lalos* Dubois, Grosjean, Ohler, Adler & Zhao (Frost 2025). Chen et al. (2018) recently synonymized all these genera under *Leptobrachella* based on a large-scale molecular phylogenetic analysis; however, these authors did not include the type species of *Leptobrachella*. The genus currently comprises 114 recognized species. Members of *Leptobrachella* inhabit the forest floor and rocky streams in hilly evergreen forests from southern China and Myanmar through mainland Indochina to peninsular Malaysia, the island of Borneo and Natuna Islands (Frost 2025). Recent studies suggested that species diversity within the genus has been significantly underestimated, with numerous new species described over the past decade (Chen et al. 2018; Hoang et al. 2019, 2024, 2025; Qian et al. 2020; Li et al. 2020; Shi et al. 2021, 2023; Luo et al. 2022, 2025; Luong et al. 2023). In Vietnam, a total of 38 species of *Leptobrachella* are currently known, and seven new taxa have been described over the past five years (Hoang et al. 2025; Frost 2025).

As a result of field surveys conducted in Dak Lak Province, Vietnam, we discovered a new population of *Leptobrachella* in the coastal forest of Deo Ca Mountain. Molecular analysis revealed that the frog population from Dak Lak Province belongs to the *L. applebyi* group, Asian leaf-litter toads with a restricted distribution in the South Central and Central Highlands of Vietnam. Closer morphological analysis revealed that this population represents an undescribed species, which we describe herein.

## ﻿Material and methods

### ﻿Sampling

**Figure 1.**
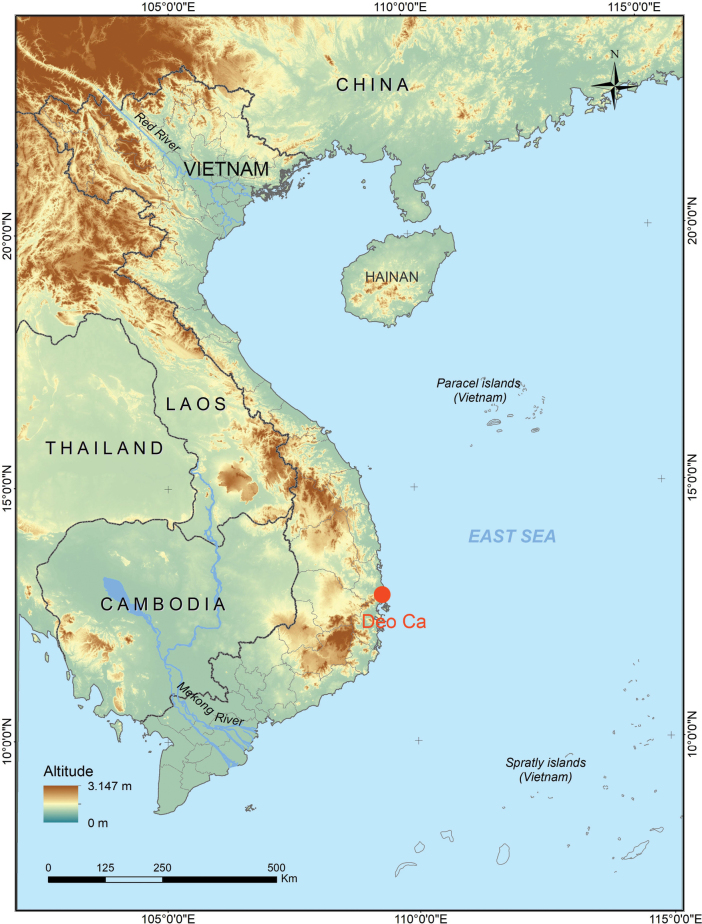
Map showing the type locality (red circle) of *Leptobrachella deocaensis* sp. nov. in Deo Ca Mountain, Dak Lak Province, Vietnam.

Field surveys were conducted in the coastal forest of Deo Ca Mountain, Dak Lak Province (formerly Phu Yen Province), Vietnam (Fig. 1) in April 2022 and in May 2023 by D.T. Do, C.V. Hoang, T.Q. Phan, H.Q. Nguyen, C.T. Pham, T.Q. Nguyen (hereafter Do et al.). Geographic coordinates and elevations were obtained by using a Garmin GPSMAP 78S satellite communicator. After live photography, frogs were anaesthetized and euthanized in a closed vessel with a piece of cotton wool containing ethyl acetate (Simmons 2002), fixed in 80% ethanol for five hours, and then later transferred to 70% ethanol for permanent storage. Tissue samples were preserved separately in 70% ethanol prior to fixation. Sex was determined by the direct observation of calling males in life or by gonadal dissection. All specimens were deposited in the collection of the Institute of Biology (IB), Hanoi, Vietnam.

### ﻿Molecular analysis

DNA was extracted from four tissue samples using PureLink™ RNA Micro Scale Kit (Thermo Fisher Scientific company), following the manufacturer’s instructions. Following Hoang et al. (2019), the target sequence comprised approximately 560 base pairs of the 16S rRNA gene from the mitochondrial genome (Suppl. material 1). Total DNA was amplified using PCR Applied Biosystems, PCR volume consisted of 25 μl, including 12 μl of Mastermix, 6 μl of water, 1 μl of each primer at concentration of 10 pmol/μl, and 5 μl of DNA. Primers used in PCR and sequencing were as follows: LR–N–13398 (5’–CGCCTGTTTACCAAAAACAT –3’; forward) and LR–J 12887 (5’–CCGGTCTGAACTCAGATCACGT –3’; reverse) (Simon et al. 1994). PCR conditions: 94 °C for 5 minutes of initial denaturation; with 35 cycles of denaturation at 94 °C for 30 s, annealing at 56 °C for 30 s, and extension at 72 °C for 45 s; and the final extension at 72 °C for 7 minutes. PCR products were sent to Apical Scientific company for sequencing (https://apicalscientific.com). Nucleotide sequences were deposited in GenBank under the accession numbers PX723918–PX723921.

### ﻿Phylogenetic analysis

In addition to the four newly collected samples, we included 112 publicly available 16S rRNA sequences for 96 species in GenBank for phylogenetic analyses (Hoang et al. 2019, 2024, 2025; Chen et al. 2020; Luong et al. 2023). Sequences of Leptobrachium cf. chapaense (Bourret) and *Xenophrys truongsonensis* Luong, Hoang, Pham, Nguyen, Orlov, Ziegler & Nguyen were included as outgroup taxa (Hoang et al. 2019, 2024, 2025; Luong et al. 2023). Locality information and accession numbers for all sequences included in the analysis are shown in Suppl. material 1. Chromas Pro software (Technelysium Pty Ltd., Tewantin, Australia) was used to edit the sequences, which were aligned using the ClustalW (Thompson et al. 1997) option in MEGA11 (Tamura et al. 2021) with default parameters and subsequently optimized manually in BioEdit ver. 7.0.5.2 (Hall 1999). We then checked the initial alignments by eye and adjusted them slightly to minimize gaps. Pairwise comparisons of uncorrected sequence divergences (p-distance) were calculated with MEGA11 (Tamura et al. 2021) when the outgroup was excluded. Variance was estimated using a bootstrap method with 1000 replicates using nucleotide substitution, while gaps/missing data were treated as pairwise deletions.

Maximum likelihood phylogenies were inferred using IQ-TREE ver. 2.3.6 (Nguyen et al. 2015) under the GTR+R4+F model for 1000 ultrafast bootstraps (Minh et al. 2013), as well as the Shimodaira-Hasegawa-like approximate likelihood-ratio test (Guindon et al. 2010). In the BI analysis, the parameters for each partition were unlinked, and branch lengths were allowed to vary proportionately across partitions. Bayesian inference phylogenies were inferred using MrBayes ver. 3.2.6 under JC+F model (2 parallel runs, 10,000,000 generations) (Ronquist et al. 2012), in which the initial 25% of sampled data were discarded as burn-in, followed by calculations of Bayesian posterior probabilities and the 50% majority-rule consensus of the post burn-in trees sampled at stationarity. We checked parameter estimates and convergence using TRACER ver. 1.7.1 (Rambaut et al. 2018). We considered Bayesian posterior probability (BPP) and ultrafast bootstrap (UFB) support values of greater than or equal to 0.95 to indicate strong support (Felsenstein 1985; Hoang et al. 2018).

### ﻿Morphological analyses

A total of 17 morphological characteristics were measured on preserved specimens using a digital caliper to the nearest 0.01 mm, following Rowley et al. (2017) and Hoang et al. (2019), as follows:

**SVL** snout-vent length;

**HL** head length from tip of snout to rear of jaws;

**HW** head width at commissure of jaws;

**SL** snout length from tip of snout to anterior corner of eye;

**ED** diameter of exposed portion of eyeball;

**IOD** interorbital distance (between the inner edges of the upper eyelids);

**TD** horizontal diameter of tympanum;

**TED** distance from anterior edge of tympanum to posterior corner of eye;

**TL** tibia length (from knee to tarsus);

**NED** distance from nostril to anterior edge of eye;

**IND** distance between nostrils;

**SND** distance from nostril to tip of snout;

**ML** manus length from tip of third digit to proximal edge of inner palmar tubercle;

**PL** pes length from tip of fourth toe to proximal edge of inner metatarsal tubercle;

**F1–3** length of fingers 1–3 from tip to distal edge of inner palmar tubercle.

Morphological comparisons between the new taxon and its congeners of the *Leptobrachella applebyi* group were based on the specimen’s examination and the following literature: Inger et al. (1999); Rowley and Cao (2009); Rowley et al. (2010a, 2010b, 2011, 2016); Poyarkov et al. (2015); Duong et al. (2018); Nguyen et al. (2018); and Hoang et al. (2025).

## ﻿Results

### ﻿Molecular phylogenetic analysis

**Figure 2.**
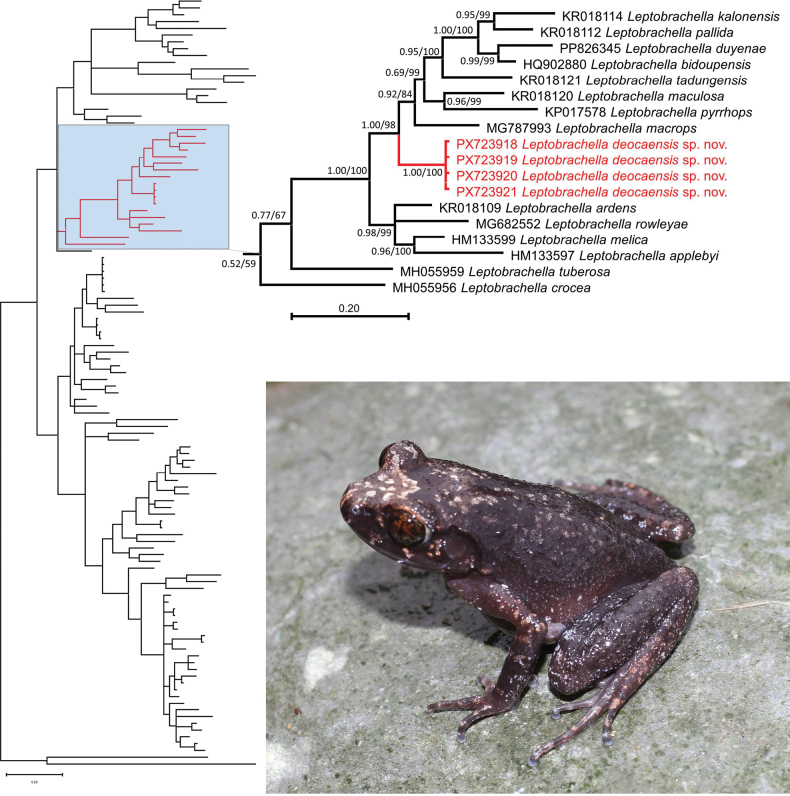
The Bayesian inference (BI) tree based on the partial 16S rRNA mitochondrial gene. Values at nodes correspond to BI/ML support values, respectively. Leptobrachium cf. chapaense and *Xenophrys truongsonensis* were used as the outgroup.

This phylogenetic analysis included 112 nucleotide sequences of 16S rRNA for *Leptobrachella* and its close relatives. Among 534 nucleotide positions 381 sites were conserved and 141 sites were variable, of which 93 were found to be potentially parsimony-informative. Evolutionary analyses were conducted in MEGA11 (Tamura et al. 2021). The estimated transition/transversion bias (R) is 2.102. Substitution pattern and rates were estimated under the Tamura (1992) model. The nucleotide frequencies are A = 31.19%, T/U = 23.81%, C = 24.57%, and G = 20.43%. Phylogenetic analyses employing ML and BI were nearly identical, with most well supported nodes on the ML tree also well supported on the Bayesian tree, and only the BI tree is presented (Fig. 2). In both analyses, the four newly collected samples were nested in a distantly related monophyletic group within *Leptobrachella* (Fig. 2). The new species is embedded in the *L. applebyi* group with strong nodal support from both analyses (1.00/98) (Fig. 2). Interspecific uncorrected p-distance of the species within *L. applebyi* group ranged from 4.50% (between *L. duyenae* Hoang, Pham, Phan, Do, Wang, Jiang & Nguyen and *L. bidoupensis* Rowley, Le, Tran & Hoang) to 14.75% (between *L. duyenae* and *L. crocea* Rowley, Hoang, Le, Dau & Cao) (Table 1). The genetic divergence between the new species and other members of the *L. applebyi* group ranged from 6.34% (*L. macrops* Duong, Do, Ngo, Nguyen & Poyarkov) to 13.20% (*L. crocea*) (Table 1).

**Table 1.** Uncorrected (“p”) distance (%) matrix showing percentage pairwise genetic divergence (16S rRNA gene) between members of the *Leptobrachella applebyi* group.

	Species	1	2	3	4	5	6	7	8	9	10	11	12	13	14	15
**1**	*Leptobrachella deocaensis* sp. nov.	**0.00**														
**2**	*L. macrops*	6.34	**0.00**													
**3**	*L. maculosa*	7.75–7.76	8.34	**0.00**												
**4**	*L. pyrrhops*	8.84–8.86	8.98	8.41	**0.00**											
**5**	*L. tadungensis*	7.13–7.14	8.44	7.96	9.86	**0.00**										
**6**	*L. kalonensis*	10.51–10.53	9.01	9.31	10.84	7.58	**0.00**									
**7**	*L. duyenae*	9.30–9.32	8.37	9.94	10.79	9.36	8.90	**0.00**								
**8**	*L. bidoupensis*	8.15–8.17	8.69	8.62	9.62	7.13	6.04	4.50	**0.00**							
**9**	*L. rowleyae*	8.50–8.52	11.15	8.97	12.00	10.17	12.08	12.05	11.75	**0.00**						
**10**	*L. pallida*	9.98–10.00	8.43	9.28	9.64	8.25	5.83	6.44	5.18	10.50	**0.00**					
**11**	*L. melica*	8.95–8.61	11.55	8.62	12.40	10.47	11.94	10.89	9.29	7.40	10.72	**0.00**				
**12**	*L. ardens*	8.13–8.15	9.26	7.32	9.17	8.90	10.76	10.84	9.51	7.38	9.11	4.89	**0.00**			
**13**	*L. applebyi*	9.59–9.61	12.07	10.09	13.48	11.12	12.79	10.77	9.89	10.13	11.11	6.47	7.35	**0.00**		
**14**	*L. tuberosa*	11.87–11.90	14.56	11.07	12.66	11.12	12.81	14.19	11.44	13.16	12.30	11.83	11.25	13.58	**0.00**	
**15**	*L. crocea*	13.17–13.20	14.62	14.60	14.42	12.93	12.32	14.75	12.07	14.61	12.39	11.72	12.41	13.43	12.30	**0.00**

We hypothesize that the molecular and morphological divergences between the new samples and other known species of *Leptobrachella* is evidence for the existence of an undescribed species, which we name below.

### ﻿Taxonomic account

#### ﻿ Leptobrachella deocaensis sp. nov.

Taxon classificationAnimaliaAnuraMegophryidae

90210196-D36B-55C9-80BB-A30E71B4A788

https://zoobank.org/1571E948-C1E4-4765-AF62-48E8D8B6AE51

Figs 3, 4; Table 2

##### Type material.

***Holotype*** • IB A.6440, adult male, collected by Do et al. on 17 May 2023, in Mui Dien coastal forest, Deo Ca Mountain (12°53'24.6"N, 109°26'33.5"E, 58 m a.s.l.), Dak Lak Province, Vietnam. ***Paratypes* (*N* = 4).** • IB A.6441–A.6444, adult females, collected by Do et al. on 14 April 2022, in Mui Dien coastal forest, Deo Ca Mountain (12°52'36.8"N, 109°26'39.1"E, 146 m a.s.l.), Dak Lak Province, Vietnam.

##### Diagnosis.

*Leptobrachella deocaensis* sp. nov. is distinguished from its congeners by a combination of the following morphological characteristics: the largest body size in the *L. applebyi* group (35.31 mm in adult male and 34.41–37.92 mm in adult females); head slightly longer than wide; tympanum distinct; dorsal skin relatively smooth with low, small round tubercles; belly grey with white dust; ventrolateral glands and femoral glands absent; toe webbing absent; and iris coppery gold.

In terms of genetic divergence, the new species is separated from its congeners by an uncorrected distance (16S rRNA) of ≥ 6.34%).

##### Description of holotype.

**Figure 3.**
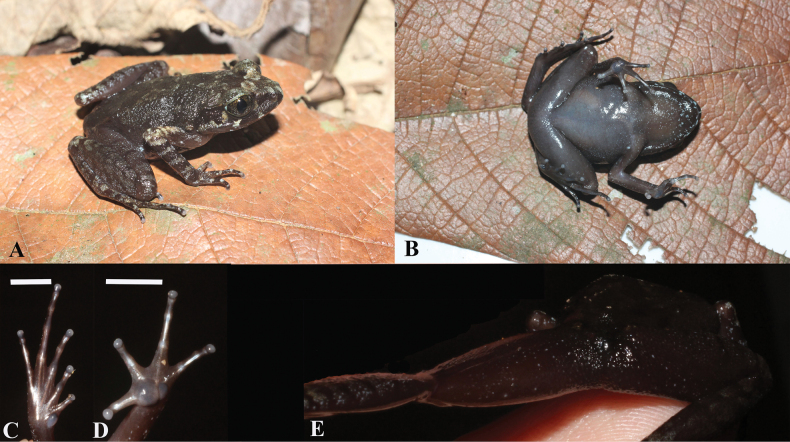
Holotype of *Leptobrachella deocaensis* sp. nov. in life (IB A.6440, male). **A.** Dorsolateral view; **B.** Ventral view; **C.** Underside of right foot; **D.** Underside of left hand; **E.** Cloacal and hamstrings area. Scale bars: 5 mm (**C, D**).

Body size large (SVL 35.31 mm); head longer than wide (HL/HW 1.04); snout obtusely pointed in dorsal view, slightly projecting beyond margin of lower jaw; nostril oval, located closer to the tip of snout than to eye (SND/NED 0.49); canthus rostralis round; loreal region sloping; interorbital space flat, slightly wider than distance between nostrils (IOD/IND 1.23); eyes large; pupil vertical; eye diameter shorter than snout length (ED/SL 0.83); tympanum distinct, round, larger than half of eye (TD/ED 0.54); tympanic rim not elevated relative to skin of temporal region; vomerine teeth absent; pineal ocellus absent; vocal sac openings present; tongue large, broad, with small notch at tip; supratympanic fold forming a distinct ridge, running from posterior corner of eye towards axillary gland (Fig. 3).

Forelimbs thin, slender; fingertips round, slightly broader than phalange width; relative finger lengths: I < II < IV < III; nuptial pad absent; subarticular tubercles absent; large round inner palmar tubercle distinctly separated from small laterally compressed outer palmar tubercle; finger webbing absent; fingers without lateral fringes (Fig. 3D).

Hindlimbs slender; tibia slightly longer than half of snout-vent length (TL/SVL = 0.51); tips of toes like those of fingers; relative toe lengths I < II < V<III < IV; subarticular tubercles absent; inner metatarsal tubercle large, oval, pronounced; outer metatarsal tubercle absent; toe webbing absent; toes without lateral fringes (Fig. 3C).

Dorsal skin relatively smooth with low, small round tubercles; dorsal surfaces of thigh, upper arm and upper eyelid with thicker small tubercles density; supra-axillary gland slightly raised (approximately 1.0 mm in diameter); around vent quite similar to the thigh area; femoral glands absent; ventral skin smooth; pectoral gland round as small as a white dot; ventrolateral glands absent (Fig. 3).

Color in life: dorsum dark brown to almost black with small, lighter mottled patches and speckles; flanks blackish grey with two white flecks; interorbital region with two symmetrical whitish grey markings; tympanum and supratympanic ridge blackish grey; upper lip with whitish grey bars; dorsal surface of limbs, fingers and toes with diffuse, transverse whitish grey bars; chin and throat grey with fine white dust; chest and belly grey with white dust scattered on the edge of belly; ventral surface of thigh and arm grey with white dust; pectoral and supra-axillary gland white; iris coppery gold (Fig. 3).

Color in preservative: dorsal surface grey to blackish grey with white patches and speckles; ventral surface grey with white dust scattered on the edge of belly; pectoral and supra-axillary gland white, indistinct in preservative.

##### Variation.

**Figure 4.**
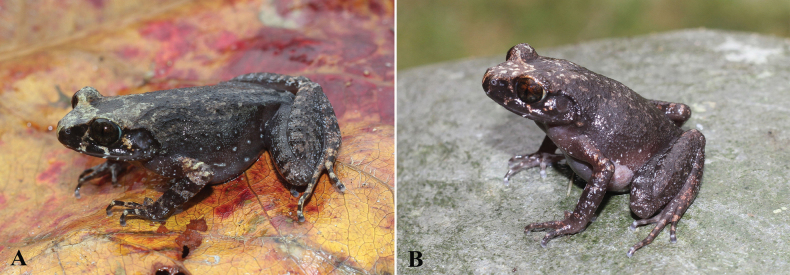
Female paratypes of *Leptobrachella deocaensis* sp. nov. in life in dorsolateral view. **A.**IB A.6441; **B.**IB A.6443.

Variation exists in body size and color pattern in life (Table 2, Figs 3, 4). Glands around the cloacal opening vary in size and number. The male holotype (IB A.6440) has a vocal sac under the throat and chin, while females do not. The female (IB A.6444) is smaller in size than the other females and the male holotype. In preservative, the dorsal skin texture varies from finely tuberculate to smooth.

**Table 2.** Measurements (in mm) and proportions of the type series of *Leptobrachella deocaensis* sp. nov. (for other abbreviations see Material and methods).

Voucher	IB A.6440	IB A.6441	IB A.6442	IB A.6443	IB A.6444
**Type status**	H	P	P	P	P
**Sex**	M	F	F	F	F
**SVL**	35.31	36.72	36.02	37.92	34.41
**HL**	14.01	15.06	14.77	16.15	13.18
**HW**	13.43	14.20	13.88	15.33	12.58
**SL**	5.06	5.65	5.24	5.90	5.03
**ED**	4.21	4.01	4.55	4.78	4.11
**IOD**	3.58	4.46	3.86	4.52	3.49
**TD**	2.27	2.26	2.48	2.08	2.45
**TED**	1.86	1.69	2.01	1.81	1.80
**TL**	18.00	18.35	18.43	19.56	17.24
**NED**	3.37	3.84	3.46	3.81	3.39
**IND**	2.92	3.39	3.22	3.46	2.29
**SND**	1.64	2.07	2.38	2.25	1.77
**ML**	9.27	9.71	9.95	10.05	9.68
**PL**	14.99	17.31	16.81	17.24	16.60
**F1**	4.98	5.02	4.53	4.83	4.69
**F2**	3.90	4.66	4.12	4.29	3.87
**F3**	5.98	7.18	6.94	7.66	6.44
**HL/HW**	1.04	1.06	1.06	1.05	1.05
**SND/NED**	0.49	0.54	0.69	0.59	0.52
**IOD/IND**	1.23	1.32	1.20	1.31	1.52
**ED/SL**	0.83	0.71	0.87	0.81	0.82
**TD/ED**	0.54	0.56	0.55	0.44	0.60
**TL/SVL**	0.51	0.50	0.51	0.52	0.50
**HL/SVL**	0.40	0.41	0.41	0.43	0.38
**TD/SVL**	0.06	0.06	0.07	0.05	0.07
**SL/SVL**	0.14	0.15	0.15	0.16	0.15

##### Etymology.

The specific name “deocaensis” derives from Deo Ca Mountain, the type locality of the new species in Dak Lak Province, Vietnam. As common names, we suggest Deoca Litter Frog (English) and Cóc mày đèo cả (Vietnamese).

##### Distribution and ecology.

**Figure 5.**
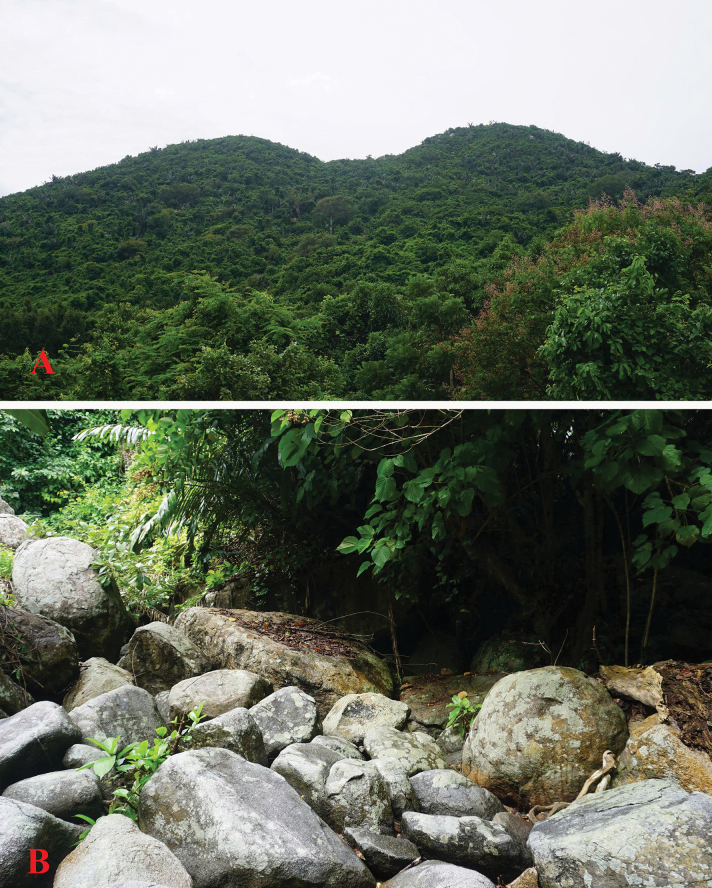
Habitat of *Leptobrachella deocaensis* sp. nov. in the Mui Dien coastal forest, Deo Ca Mountain, Dak Lak Province, Vietnam. **A.** Evergreen forest; **B.** Microhabitat.

*Leptobrachella deocaensis* sp. nov. is currently known from Mui Dien coastal forest of Deo Ca Mountain, Dak Lak Province, Vietnam. The type specimens were found on rocks or on ground in a rocky stream in the coastal forest at elevations between 50 m and 150 m a.s.l. (Fig. 5). The surrounding habitat was secondary evergreen forest of medium, small hardwood and shrubs. Air temperatures at the sites ranged from 26.8–33.5 °C and relative humidity was 60–78%. *Leptobrachella deocaensis* sp. nov. occurs sympatrically with other amphibian species found at the site, including *Ingerophrynus galeatus* (Günther); *Microhyla mukhlesuri* Hasan, Islam, Kuramoto, Kurabayashi & Sumida; *Limnonectes phuyenensis* Pham, Do, Le, Ngo, Nguyen, Ziegler & Nguyen; *Hylarana annamitica* Sheridan & Stuart; and *Polypedates mutus* (Smith).

##### Comparisons.

We compared the new species with other members of the *Leptobrachella applebyi* group based on data obtained from the literature (Suppl. material 2).

*Leptobrachella deocaensis* sp. nov. is morphologically distinguishable from the 14 recognized members of the *L. applebyi* group by having the largest body size (35.31 mm in adult male and 34.41–37.92 mm in adult females) (Suppl. material 2).

The new species is most closely related to *L. macrops*, from which it can be distinguished by significant genetic divergence (p = 6.34%; Fig. 2; Table 1). Morphologically, *Leptobrachella deocaensis* sp. nov. differs from *L. macrops* by having the dorsal skin relatively smooth with low, small round tubercles (vs roughly granular with larger tubercles in *L. macrops*), the absence of black spots on flanks (vs present in *L. macrops*), toes without webbing (vs rudimentary in *L. macrops*) and iris coppery gold (vs bright orange-gold with greenish tint in lower half and fine black reticulations throughout in *L. macrops*);

from *L. bidoupensis* by having a belly grey with white dust (vs dark brownish red with white speckling in *L. bidoupensis*), indistinct dorsolateral markings (vs distinct in *L. bidoupensis*), the absence of black spots on flanks (vs present in *L. bidoupensis*), toes without webbing (vs rudimentary in *L. bidoupensis*), toes without lateral fringes (vs narrow in *L. bidoupensis*), and iris coppery gold (vs upper half coppery red, fading to pale silver in lower half in *L. bidoupensis*);

from *L. tadungensis* by having a belly grey with white dust (vs dark brownish with fine white speckling in *L. tadungensis*), the absence of black spots on flanks (vs present in *L. tadungensis*), and indistinct dorsolateral markings (vs distinct in *L. tadungensis*);

from *L. pallida* by having dorsal skin relatively smooth with low, small round tubercles scattered (vs skin on dorsum coarsely shagreened in *L. pallida*), belly grey with white dust (vs dark brownish red with faint white speckling in *L. pallida*), the absence of black spots on flanks (vs present in *L. pallida*), indistinct dorsolateral markings (vs distinct in *L. pallida*), and iris coppery gold (vs copper in upper half, gold in lower half in *L. pallida*).

from *L. applebyi* by having toes without lateral fringes (vs narrow in *L. applebyi*), belly grey with white dust (vs dark brownish pink with white speckling in *L. applebyi*), indistinct dorsolateral markings (vs distinct in *L. applebyi*), the absence of black spots on flanks (vs present in *L. applebyi*), and toes without webbing (vs rudimentary in *L. applebyi*);

from *L. ardens* by having a belly grey with white dust (vs dark brownish red with white speckling in *L. ardens*), indistinct dorsolateral markings (vs distinct in *L. ardens*), black spots on flanks absent (vs present in *L. ardens*), and iris coppery gold (vs brown in *L. ardens*);

from *L. crocea* by having dorsal skin relatively smooth with low, small round tubercles scattered (vs highly tuberculate in *L. crocea*), belly grey with white dust (vs bright orange in *L. crocea*), toes without webbing (vs rudimentary in *L. crocea*), and iris coppery gold (vs pale gold in *L. crocea*);

from *L. duyenae* by having dorsal skin relatively smooth with low, small round tubercles scattered (vs coarsely shagreened on dorsum with scattered, low, small tubercles in *L. duyenae*), belly grey with white dust (vs dark brownish to grey whitish with white speckling in *L. duyenae*), indistinct dorsolateral markings (vs distinct in *L. duyenae*), the absence of black spots on flanks (vs present in *L. duyenae*), and iris coppery gold (vs iris bicolored, upper half golden, lower half silver in *L. duyenae*);

from *L. kalonensis* by having a belly grey with white dust (vs pale brownish pink with white speckling in *L. kalonensis*), indistinct dorsolateral markings (vs distinct in *L. kalonensis*), the absence of black spots on flanks (vs present in *L. kalonensis*), and iris coppery gold (vs iris gold, coppery orange in upper third in *L. kalonensis*);

from *L. maculosa* by having a belly grey with white dust (vs dark brownish with white speckling in *L. maculosa*), indistinct dorsolateral markings (vs distinct in *L. maculosa*), the absence of black spots on flanks (vs present in *L. maculosa*), and iris coppery gold (vs iris copper in upper half and gold in lower half in *L. maculosa*);

from *L. melica* by having a belly grey with white dust (vs white to pale pink with diffuse dark brown blotches and white speckling in *L. melica*), indistinct dorsolateral markings (vs distinct in *L. melica*), the absence of black spots on flanks (vs present in *L. melica*), toes without webbing (vs rudimentary in *L. melica*), and iris coppery gold (vs iris dark gold in *L. melica*);

from *L. pyrrhops* by having dorsal skin relatively smooth with low, small round tubercles scattered (vs slightly shagreened in *L. pyrrhops*), belly grey with white dust (vs grey pinkish to dark brownish-violet in *L. pyrrhops*), the absence of black spots on flanks (vs present in *L. pyrrhops*), toes without webbing (vs rudimentary in *L. pyrrhops*), and iris coppery gold (vs iris bicolored: bright orange-gold in upper half, fading to greenish-silver in lower third in *L. pyrrhops*);

from *L. rowleyae* by having a belly grey with white dust (vs pinkish milk-white with dense whitish speckling evenly scattered on entire ventral surface in *L. rowleyae*), the absence of black spots on flanks (vs present in *L. rowleyae*), toes without webbing (vs rudimentary in *L. rowleyae*), and iris coppery gold (vs iris bicolored, copper-orange in upper half, fading to golden in lower third in *L. rowleyae*);

from *L. tuberosa* by having dorsal skin relatively smooth with low, small round tubercles scattered (vs highly tuberculate in *L. tuberosa*), belly grey with white dust (vs white with small grey spots/streaks in *L. tuberosa*), and toes without webbing (vs rudimentary in *L. tuberosa*).

## ﻿Discussion

The discovery of *Leptobrachella deocaensis* brings the total number of known species in the genus to 115 and the species known from Vietnam to 39 (Hoang et al. 2025). It is likely that the number of recorded species in this genus will continue to increase as a result of additional fieldwork in poorly studied areas and the implementation of molecular techniques to delineate species (Rowley et al. 2017; Hoang et al. 2025).

Although the species in the *L. applebyi* group have narrow and localized distribution ranges, restricted to the South Central and Central Highlands of Vietnam, the genetic divergence between *Leptobrachella deocaensis* and other members of *Leptobrachella* was at least 6.34% (16S rRNA gene). This indicates a profound genetic separation of *L. deocaensis* from other species in the *L. applebyi* group, and highlights a strong association between this divergence and the unique environmental conditions at Deo Ca Mountain.

*Leptobrachella deocaensis* is geographically closest to *L. duyenae* and *L. macrops*, with distances of approximately 28 km and 67 km, respectively. However, *Leptobrachella deocaensis* was found on rocks or on the ground along a rocky stream in coastal forest at low elevations between 50 m and 150 m a.s.l., whereas *L. duyenae* was collected in montane evergreen forest at a higher elevation (822 m a.s.l.) and *L. macrops* was recorded in evergreen forests at elevations between 471 m and 630 m a.s.l. Despite their geographic proximity, these three species occupy distinct habitats at different elevation levels, and their distribution ranges do not overlap.

*Leptobrachella deocaensis* seems to be endemic to the Mui Dien coastal forest, a habitat characterized by extremely harsh coastal conditions. Our surveys in the surrounding areas, including both hillsides of the Deo Ca Mountain, did not yield any individuals in the adjacent evergreen forest. Nevertheless, the coastal forest remains poorly studied, highlighting the urgent need to assess potential threats to the population of *L. deocaensis* within its restricted range.

## Supplementary Material

XML Treatment for Leptobrachella deocaensis

## ﻿Additional information

### Use of AI

No use of AI was reported.

### Author ORCIDs

Dang Trong Do https://orcid.org/0000-0003-3048-1434

Truong Quang Nguyen https://orcid.org/0000-0002-6601-0880

Chung Van Hoang https://orcid.org/0000-0002-0709-974X

Thomas Ziegler https://orcid.org/0000-0002-4797-609X

Cuong The Pham https://orcid.org/0000-0001-5158-4526

### Data availability

All of the data that support the findings of this study are available in the main text or Supplementary Information.

## Supplementary materials

Supplementary material 1 GenBank accession numbers Data type docx Explanation note GenBank accession numbers and associated samples used in this study. https://binary.pensoft.net/file/1516182This dataset is made available under the Open Database License (http://opendatacommons.org/licenses/odbl/1.0/). The Open Database License (ODbL) is a license agreement intended to allow users to freely share, modify, and use this Dataset while maintaining this same freedom for others, provided that the original source and author(s) are credited.Dang Trong Do, Truong Quang Nguyen, Chung Van Hoang, Thomas Ziegler, Cuong The Pham

Supplementary material 2 Diagnostic characteristics Data type docx Explanation note Selected diagnostic characteristics for the species in the *Leptobrachella applebyi* group (modified from Rowley et al. 2017b; Hoang et al. 2025). NA = not available. https://binary.pensoft.net/file/1516183This dataset is made available under the Open Database License (http://opendatacommons.org/licenses/odbl/1.0/). The Open Database License (ODbL) is a license agreement intended to allow users to freely share, modify, and use this Dataset while maintaining this same freedom for others, provided that the original source and author(s) are credited.Dang Trong Do, Truong Quang Nguyen, Chung Van Hoang, Thomas Ziegler, Cuong The Pham

## References

[B1] Chen JM Poyarkov Jr NA Suwannapoom C Lathrop A Wu YH Zhou WW Yuan ZY Jin JQ Chen HM Liu HQ Nguyen TQ Nguyen SN Duong TV Eto K Nishikawa K Matsui M Orlov NL Stuart BL Brown RM Rowley JJL Murphy RW Wang YY Che J (2018) Large-scale phylogenetic analyses provide insights into unrecognized diversity and historical biogeography of Asian leaf-litter frogs, genus *Leptolalax* (Anura: Megophryidae). Molecular Phylogenetics and Evolution 124: 162–171. 10.1016/j.ympev.2018.02.020 29530499

[B2] Chen JM Xu K Poyarkov NA Wang K Yuan ZY Hou M Suwannapoom C Wang J Che J (2020) How little is known about “the little brown frogs”: description of three new species of the genus *Leptobrachella* (Anura: Megophryidae) from Yunnan Province, China. Zoological Research 41: 1–22. 10.24272/j.issn.2095-8137.2020.036 32323508 PMC7231475

[B3] Duong TV Do DT Ngo CD Nguyen TQ Poyarkov NA (2018) A new species of the genus *Leptolalax* (Anura: Megophryidae) from southern Vietnam. Zoological Research 39: 181–196. 10.24272/j.issn.2095-8137.2018.009 PMC5968861 29643325

[B4] Felsenstein J (1985) Confidence limits on phylogenies: An approach using the bootstrap. Evolution; International Journal of Organic Evolution 39(4): 783–791. 10.1111/j.1558-5646.1985.tb00420.x 28561359

[B5] Frost DR (2025) Amphibian Species of the World: an on-line reference. Version 6.2. American Museum of Natural History, New York, USA. https://amphibiansoftheworld.amnh.org/Amphibia/ [accessed on 9 August 2025]

[B6] Guindon S Dufayard JF Lefort V Anisimova M Hordijk W Gascuel O (2010) New algorithms and methods to estimate maximum-likelihood phylogenies: Assessing the performance of PhyML 3.0. Systematic Biology 59(3): 307–321. 10.1093/sysbio/syq010 20525638

[B7] Hall TA (1999) BioEdit: a user-friendly biological sequence alignment editor and analysis program for Windows 95/98/NT. Nucleic Acids Symposium 41: 95–98.

[B8] Hoang DT Chernomor O von Haeseler A Minh BQ Vinh LS (2018) UFBoot2: Improving the ultrafast bootstrap approximation. Molecular Biology and Evolution 35(2): 518–522. 10.1093/molbev/msx281 29077904 PMC5850222

[B9] Hoang CV Nguyen TT Luu VQ Nguyen TQ Jiang JP (2019) A new species of *Leptobrachella* Smith 1925 (Anura: Megophryidae) from Thanh Hoa Province, Vietnam. The Raffles Bulletin of Zoology 67: 536–556.

[B10] Hoang CV Luong AM Nguyen TQ Nguyen TT Ninh HT Le LTH Ziegler T Pham CT (2024) A new species of *Leptobrachella* Smith 1925 (Anura, Megophryidae) from Lai Chau Province, Vietnam. Biodiversity Data Journal 12: e136491. 10.3897/BDJ.12.e136491 PMC11555430 39534725

[B11] Hoang VC Pham TC Phan QT Do TD Wang B Jiang JP Nguyen QT (2025) Hidden biodiversity in the tropical rain forests: two new species of *Leptobrachella* Smith 1925 (Anura: Megophryidae) from Vietnam. Asian Herpetological Research 16(2): 1–23. 10.3724/ahr.2095-0357.2024.0039

[B12] Inger RF Orlov N Darevsky I (1999) Frogs of Vietnam: A report on new collections. Fieldiana. Zoology 92: 1–46.

[B13] Li SZ Liu J Wei G Wang B (2020) A new species of the Asian leaf litter toad genus *Leptobrachella* (Amphibia, Anura, Megophryidae) from southwest China. ZooKeys 943: 91–118. 10.3897/zookeys.943.51572 32624677 PMC7324409

[B14] Luo T Wang W Peng D Lei B Deng H Ji S Huang H Zhou J (2022) A new species of the Asian leaf litter toad genus *Leptobrachella* (Amphibia, Anura, Megophryidae) from Chongqing City, Southwest China. Asian Herpetological Research 13: 75–95. 10.16373/j.cnki.ahr.210052

[B15] Luo T Zhao ZF Wang ZL Xiao MY Deng H Xiao N Zhou J (2025) Diversification outbreaks and dynamics of Asian leaf-litter frogs, genus *Leptobrachella* (Anura, Megophryidae), with the description of a new species from Guizhou Province, China. Zoosystematics and Evolution 101(1): 223–243. 10.3897/zse.101.137392

[B16] Luong AM Hoang CV Pham CT Ziegler T Nguyen TQ (2023) Two new species of *Leptobrachella* Smith 1925 (Amphibia: Megophryidae) from Cao Bang Province, Vietnam. Zootaxa 5369(3): 301–335. 10.11646/zootaxa.5369.3.1 38220711

[B17] Minh BQ Nguyen MA von Haeseler A (2013) Ultrafast approximation for phylogenetic bootstrap. Molecular Biology and Evolution 30(5): 1188–1195. 10.1093/molbev/mst024 23418397 PMC3670741

[B18] Nguyen LT Schmidt HA von Haeseler A Minh BQ (2015) IQ-TREE: A fast and effective stochastic algorithm for estimating maximum-likelihood phylogenies. Molecular Biology and Evolution 32(1): 268–274. 10.1093/molbev/msu300 25371430 PMC4271533

[B19] Nguyen TL Poyarkov NA Le TD Vo BD Ninh TH Duong VT Murphy RW Sang NV (2018) A new species of *Leptolalax* (Anura: Megophryidae) from Son Tra Peninsula, central Vietnam. Zootaxa 4388(1): 1–21. 10.11646/zootaxa.4388.1.1 29690461

[B20] Poyarkov NA Rowley JJL Gogoleva SI Vassilieva AB Galoyan EA Orlov NL (2015) A new species of *Leptolalax* (Anura: Megophryidae) from the western Langbian Plateau, southern Vietnam. Zootaxa 3931(2): 221–252. 10.11646/zootaxa.3931.2.3 25781823

[B21] Qian TY Xia X Cao Y Xiao NW Yang DD (2020) A new species of *Leptobrachella* (Anura: Megophryidae) from Wuling Mountains in Hunan Province, China. Zootaxa 4816(4): 491–526. 10.11646/zootaxa.4816.4.4 33055685

[B22] Rambaut A Drummond AJ Xie D Baele G Suchard MA (2018) Posterior summarisation in Bayesian phylogenetics using Tracer 1.7. Systematic Biology 67(5): 901–904. 10.1093/sysbio/syy032 29718447 PMC6101584

[B23] Ronquist F Teslenko M van der Mark P Ayres DL Darling A Höhna S Larget B Liu L Suchard MA Huelsenbeck JP (2012) MrBayes 3.2: Efficient Bayesian phylogenetic inference and model choice across a large model space. Systematic Biology 61(3): 539–542. 10.1093/sysbio/sys029 22357727 PMC3329765

[B24] Rowley JJ Cao TT (2009) A new species of *Leptolalax* (Anura: Megophryidae) from central Vietnam. Zootaxa 2198(1): 51–60. 10.11646/zootaxa.2198.1.5 28610262

[B25] Rowley JJ Hoang HD Le DTT Dau VQ Cao TT (2010a) A new species of *Leptolalax* (Anura: Megophryidae) from Vietnam and further information on *Leptolalax tuberosus*. Zootaxa 2660(1): 33–45. 10.11646/zootaxa.2660.1.3

[B26] Rowley JJ Stuart BL Neang T Emmett DA (2010b) A new species of *Leptolalax* (Anura: Megophryidae) from northeastern Cambodia. Zootaxa 2567(1): 57–68. 10.11646/zootaxa.2567.1.3 26624626

[B27] Rowley JJ Le TTD Tran DTA Hoang HD (2011) A new species of *Leptolalax* (Anura: Megophryidae) from southern Vietnam. Zootaxa 2796(1): 15–28. 10.11646/zootaxa.2796.1.2

[B28] Rowley JJ Tran DTA Le TTD Dau VQ Peloso PLV Nguyen TQ Hoang HD Nguyen TT Ziegler T (2016) Five new, microendemic Asian Leaf-litter Frogs (*Leptolalax*) from the southern Annamite mountains, Vietnam. Zootaxa 4085(1): 63–102. 10.11646/zootaxa.4085.1.3 27394289

[B29] Rowley JJ Dau VQ Hoang H Le DT Cutajar TP Nguyen TT (2017) A new species of *Leptolalax* (Anura: Megophryidae) from northern Vietnam. Zootaxa 4243(3): 544–564. 10.11646/zootaxa.4243.3.7 28610143

[B30] Shi SC Hou Y Song Z Jiang JP Wang B (2021) A new leaf litter toad of *Leptobrachella* Smith 1925 (Anura, Megophryidae) from Sichuan Province, China, with supplementary description of *L. oshanensis*. Asian Herpetological Research 12: 143–166. 10.16373/j.cnki.ahr.200118

[B31] Shi SC Shen T Wang X Jiang JP Wang B (2023) Multiple data sources reveal a new Asian leaf litter toad of *Leptobrachella* Smith 1925 (Anura, Megophryidae) from southwestern China. Asian Herpetological Research 14: 65–94. 10.16373/j.cnki.ahr.220065

[B32] Simmons JE (2002) Herpetological collecting and collections management. Revised edition. Society for the Study of Amphibians and Reptiles, Herpetological Circular 31: 1–153.

[B33] Simon C Frati F Beckenbach A Crespi B Liu H Flook P (1994) Evolution, weighting, and phylogenetic utility of mitochondrial gene sequences and a compilation of conserved polymerase chain reaction primers. Annals of the Entomological Society of America 87(6): 651–701. 10.1093/aesa/87.6.651

[B34] Smith MA (1925) Contributions to the herpetology of Borneo. Sarawak Museum Journal 3: 15–34.

[B35] Tamura K (1992) Estimation of the number of nucleotide substitutions when there are strong transition–transversion and G + C–content biases. Molecular Biology and Evolution 9: 678–687. 1630306 10.1093/oxfordjournals.molbev.a040752

[B36] Tamura K Stecher G Kumar S (2021) MEGA 11: Molecular evolutionary genetics analysis version 11. Molecular Biology and Evolution 38(7): 3022–3027. 10.1093/molbev/msab120 33892491 PMC8233496

[B37] Thompson JD Gibson TJ Plewniak F Jeanmougin F Higgins DG (1997) The CLUSTAL_X windows interface: Flexible strategies for multiple sequence alignment aided by quality analysis tools. Nucleic Acids Research 25(24): 4876–4882. 10.1093/nar/25.24.4876 9396791 PMC147148

