# Supplementary material for: ﻿A new species of Leptobrachella Smith, 1925 (Anura, Megophryidae) from the coastal forest of Dak Lak Province, Vietnam
Source: Zookeys. 2026 Jan 20;1267:15–30. doi: 10.3897/zookeys.1267.177118 (PMC12848511; doi:10.3897/zookeys.1267.177118)
Supplement: Supplementary material 1 — GenBank accession numbers [file zookeys-1267-015_article-177118__-s001.docx]

**Supplementary material 2.** Selected diagnostic characteristics for the species in the *Leptobrachella applebyi* group (modified from Rowley et al. 2017b; Hoang et al. 2025). NA = not available

| **No** | **Species** | **Male SVL (mm)** | **Female SVL (mm)** | **Black spots on flanks** | **Bicolored**  **iris** | **Distinct dorsolateral markings** | **Toes webbing** | **Fringes on toes** | **Ventral coloration** | **Dorsal skin texture** | **References** |
| --- | --- | --- | --- | --- | --- | --- | --- | --- | --- | --- | --- |
| 1 | *Leptobrachella deocaensis* sp. nov. | 35.3 | 34.4–37.9 | Absent | Absent | No | Absent | Absent | Belly grey with white dust | Dorsal skin relatively smooth with low, round small tubercles scattered | This study |
| 2 | *L. applebyi* | 19.6–22.3 | 21.7 | Present | Absent | Yes | Rudimentary | Absent | Dark brownish pink with white speckling | Smooth | Rowley and Cao, 2009 |
| 3 | *L. ardens* | 21.3–24.7 | 25.4 | Present | Absent | Yes | Absent | Absent | Dark brownish red with white speckling | Smooth- finely shagreened | Rowley et al. 2016 |
| 4 | *L. bidoupensis* | 18.5–25.4 | 29.2–29.4 | Present | Present | Yes | Rudimentary | Narrow | Dark brownish red with white speckling | Smooth | Rowley et al. 2011 |
| 5 | *L. crocea* | 22.2–27.3 | NA | Absent | Absent | No | Rudimentary | Absent | Bright orange | Highly tuberculate | Rowley et al. 2010a |
| 6 | *L. duyenae* | 25.1–29.3 | 30.8 | Present | Present | Yes | Absent | Absent | Dark brownish to grey whitish ventral surface with white speckling on ventral surfaces of belly | Skin coarsely shagreened on dorsum with scattered, low, small tubercles | Hoang et al. 2025 |
| 7 | *L. kalonensis* | 25.8–30.6 | 28.9–30.6 | Present | Present | Yes | Absent | Absent | Pale brownish pink with white speckling | Smooth | Rowley et al. 2016 |
| 8 | *L. macrops* | 28.0–29.3 | 30.3 | Present | Present | No | Rudimentary | Absent | Greyish-violet with white speckling | Roughly granular with larger tubercles | Duong et al. 2018 |
| 9 | *L. maculosa* | 24.2–26.6 | 27 | Present | Present | Yes | Absent | Absent | Dark brownish with white speckling | Mostly smooth | Rowley et al. 2016 |
| 10 | *L. melica* | 19.5–22.7 | NA | Present | Absent | Yes | Rudimentary | Absent | White to pale pink with diffuse dark brown blotches and white speckling | Smooth | Rowley et al. 2010b |
| 11 | *L. pallida* | 24.5–27.7 | NA | Present | Present | Yes | Absent | Absent | Dark brownish red with faint white speckling | Skin on dorsum coarsely shagreened | Rowley et al. 2016 |
| 12 | *L. pyrrhops* | 30.8–34.3 | 30.8–34.3 | Present | Present | Yes | Rudimentary | Absent | Grey pinkish to dark brownish -violet | Slightly shagreened | Poyarkov et al. 2015 |
| 13 | *L.* *rowleyae* | 23.4–25.4 | 27.0–27.8 | Present | Present | No | Rudimentary | Absent | Pinkish milk-white with dense whitish speckling evenly scattered on entire ventral surface | Mostly smooth with numerous tiny tubercles and pustules finely | Nguyen et al. 2018 |
| 14 | *L. tadungensis* | 23.3–28.2 | 32.1 | Present | Absent | Yes | Absent | Absent | Dark brownish with white speckling | Smooth | Rowley et al. 2016 |
| 15 | *L. tuberosa* | 24.4–29.5 | 30.2 | Absent | Absent | No | Rudimentary | Absent | White with small grey spots/streaks | Highly tuberculate | Inger et al. 1999; Rowley et al. 2010a |
